# ‘*Candidatus* Liberibacter asiaticus’ secretory protein SDE5640 activates host autophagy dependent on PSMD10 to mediate PBA1 degradation

**DOI:** 10.1111/tpj.71147

**Published:** 2026-10-07

**Authors:** Yalin Mei, Xinyi Deng, Zaiyu Yang, Shimin Fu, Changyong Zhou, Xuefeng Wang

**Affiliations:** ^1^ Integrative Science Center of Germplasm Creation in Western China (CHONGQING) Science City, Citrus Research Institute Southwest University Chongqing 400712 China; ^2^ National Citrus Engineering Research Center, Citrus Research Institute Southwest University Chongqing 400712 China

**Keywords:** Huanglongbing, effector, autophagy, proteasome, CsPSMD10, CsPBA1

## Abstract

The ubiquitin proteasome system (UPS) and autophagy cooperatively orchestrate plant immune homeostasis, while diverse phytopathogens deploy secreted effectors to disrupt their crosstalk for successful colonization. Proteaphagy, the autophagic turnover of intact 26S proteasome holoenzymes, is a conserved stress response co‐opted by *Pseudomonas syringae* effector HopM1 to cripple host proteolysis. However, how the Huanglongbing (HLB) pathogen ‘*Candidatus* Liberibacter asiaticus’ (*C*Las) simultaneously manipulates UPS and autophagy to subvert citrus immunity remains largely uncharacterized. Here, we functionally characterize the secreted *C*Las effector SDE5640, which exacerbates HLB susceptibility in sweet orange (*Citrus sinensis*). SDE5640 directly binds the 19S proteasome chaperone CsPSMD10, whose homodimerization via Cys163/Cys190 disulfide bonds is required for immune suppression. CsPSMD10 overexpression enhances citrus susceptibility to *C*Las, whereas the dimerization‐deficient CsPSMD10‐C190S mutant confers robust HLB resistance. Mechanistically, CsPSMD10 targets CsATG8 isoforms for UPS‐dependent degradation to restrain autophagic flux; SDE5640 competitively occupies the Ank2/Ank3 domains of CsPSMD10 to disrupt CsPSMD10‐CsATG8c complexes, thereby stabilizing CsATG8c and triggering autophagy dependent on CsPSMD10. Moreover, SDE5640‐induced autophagy drives selective autophagic degradation of the 20S proteasome catalytic subunit CsPBA1, which ultimately disrupts cellular proteasome activity and attenuates salicylic acid‐mediated citrus immunity. Collectively, our findings identify a novel SDE5640‐CsPSMD10‐CsATG8c‐CsPBA1 regulatory cascade, illustrating how a single *C*Las effector synchronously rewires the UPS‐autophagy crosstalk to facilitate pathogen proliferation.

## Significance Statement

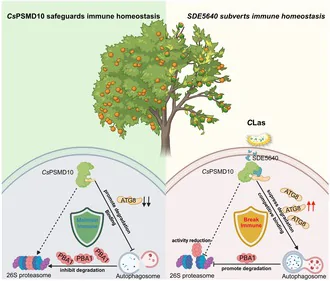

Huanglongbing severely threatens citrus production, yet how *C*Las single effector synchronously manipulates UPS‐autophagy crosstalk remains unclear. This work reveals that SDE5640 hijacks CsPSMD10 to stabilize CsATG8c and trigger autophagic breakdown of proteasome subunit PBA1, uncovering a unique plant‐specific immune regulatory module and novel molecular targets for HLB resistance breeding.

## INTRODUCTION

The ubiquitin proteasome system (UPS) and autophagy are two indispensable intracellular machineries that are responsible for removing or switching out dated metabolic pathways in all eukaryotic cells upon various internal and external cues (Pohl & Dikic, 2019). Autophagy is a major pathway of eukaryotic degradation and recycling that directs the bulk or selective phagocytosis of cytoplasmic contents into double‐membrane vesicles, called autophagosomes, for subsequent turnover (Klionsky & Codogno, 2013). The UPS is composed of many members, which can be temporarily and spatially separated into two subsystems that function in tandem for protein ubiquitylation and degradation (Vierstra, 2009). The UPS and autophagy synergistically maintain cellular and protein homeostasis. In yeast, the proteasome inhibitor MG132 impairs proteasomal activity, and the inactive proteasome becomes ubiquitinated. It is subsequently recognized by the adaptor protein Cue5 with the assistance of the molecular chaperone Hsp42. This complex further associates with ATG8 to undergo Cue5‐dependent autophagic degradation (Marshall et al., 2016). Marshall et al., identified the ubiquitin‐binding subunit of the regulatory particle non‐ATPase subunit 10 (RPN10) as a specific receptor for autophagic degradation of proteasomes (proteaphagy), triggered by either chemical or genetic inhibition of the proteasome (Marshall et al., 2015).

The UPS and autophagy serve as conserved cellular defense mechanisms in diverse organisms, playing a critical role in limiting pathogen infection. As counter defenses, pathogens have evolved strategies to manipulate the two pathways to facilitate their infection（Huang & Brumell, 2014 . For instance, *Xanthomonas campestris* pv. *vesicatoria* (Xcv) effector XopL interacts with and degrades the autophagy component SH3P2 via its E3 ligase activity to promote infection (Leong et al., 2022). The bacterial effector SopF inhibits bacterial autophagy by preventing the V‐ATPase‐ATG16L1 interaction to promote *Salmonella typhimurium* proliferation (Xu et al., 2019). The effector HopM1 of *Pseudomonas syringae* pv. *tomato* DC3000 (*Pst* DC3000) induces autophagy, increases autophagic flux and promotes bacterial proliferation in *Arabidopsis thaliana* (Üstün et al., 2018). *Pst* DC3000 effector HrpZ1 undergoes a calcium‐dependent conformation change to enhance autophagy through the modulation of ATG8 cleavage by ATG4 (Lal et al., 2020). The *C*Las effector protein SDE5 facilitates the degradation of the transcription factor MYC2 by enhancing the enzymatic activity of E3 ligase PUB21. This process inhibits the jasmonic acid (JA)‐mediated defense response and promotes pathogen infection (Zhao et al., 2025). The E3 ligase OsRGLG5 is targeted by the AvrPi9 effector from *Magnaporthe oryzae* and serves a vital role in enhancing basal resistance to rice blast (Liu et al., 2023). *Ralstonia solanacearum* effector protein RipAC modulates the activity of the immune kinase BIK1 by targeting the E3 ligase PUB4 within host cells, thereby facilitating infection (Yu et al., 2022). So far, how phytopathogen effectors co‐regulate host autophagy and the UPS remains poorly understood, and only a few cases have been documented. For instance, *Pst* DC3000 activates type III effector (T3E)‐dependent autophagy to promote the proteaphagy, facilitating bacterial proliferation (Üstün et al., 2018).

Citrus is top‐ranked in importance among all fruit crops. Citrus Huanglongbing, caused by ‘*Candidatus* Liberibacter asiaticus’ (*C*Las), is the most destructive disease that threatens the sustainability of the global citrus industry (Bastianel et al., 2005; Jagoueix et al., 1997). In citrus, accumulating evidence has demonstrated that *C*Las effector proteins modulate host autophagy and ubiquitination pathways to promote bacterial infection. For example, *C*Las effector protein SDE4580 targets the SNARE protein CsVTI1a to suppress autophagosome‐vacuole fusion, thereby promoting bacterial colonization (Cui et al., 2025). The *C*Las secretory protein SDE3 suppresses host autophagy by promoting the degradation of ATG8s, thereby aggravating HLB development in citrus (Shi, Yang, et al., 2023). Another effector, SDE4405, interacts with ATG8s proteins to activate autophagy and enhance bacterial infection (Shi, Gong, et al., 2023). The *C*Las effector SDE5 promotes pathogen proliferation by strengthening the interaction between PUB21 and MYC2, leading to suppression of jasmonic acid (JA)‐mediated defense responses (Zhao et al., 2025). Recently, it was reported that a *C*Las effector, SDE5640, downregulates salicylic acid (SA) levels in transgenic citrus shoots, thereby attenuating host immune responses. Specifically, SDE5640 suppresses proteasome activity to facilitate bacterial infection; In turn, the host proteasome mediates the degradation of this effector (Mei et al., 2025). Yet the molecular mechanism by which SDE5640 impairs proteasome function remains elusive.

In this study, we demonstrated that SDE5640 physically interacts with CsPSMD10 which is a non‐ATPase subunit of the 26S proteasome and typically functions as a chaperone. Notably, CsPSMD10 mediates the degradation of CsATG8c through the 26S proteasome system. SDE5640 significantly mitigates the degradation of CsATG8c by inhibiting the CsPSMD10‐CsATG8c interaction. Furthermore, our findings revealed that SDE5640 activates autophagy and enhances the autophagic removal of PBA1, thereby impairing proteasome activity and promoting bacterial proliferation.

## RESULTS

### SDE5640 suppresses proteasome activity and promotes *C*Las proliferation

To determine whether SDE5640 is involved in promoting *C*Las colonization, we expressed SDE5640 in ‘Wanjincheng’ orange via stable *Agrobacterium tumefaciens*‐mediated transformation. Two independent *SDE5640*‐expressing citrus lines (*SDE5640*‐1 and *SDE5640*‐2) were obtained and confirmed by RT‐PCR, β‐glucuronidase (GUS) histochemical staining and RT‐qPCR (Figure 1A; Figure S1A,B). *SDE5640*‐transgenic citrus lines and WT control plants were graft‐inoculated with *C*Las‐infected Guanximiyou pummelo (*C. grandis*) buds at 6 months (Figure 1B). The *SDE5640*‐transgenic citrus plants significantly increased the *C*Las titers, as shown by qPCR (Figure 1C). We then challenged two *SDE5640*‐transgenic ‘Wanjincheng’ orange with *Xanthomonas citri* subsp. *citri* (*Xcc*) using the pinprick method. Upon inoculation with *Xcc*, the appearance of canker‐like lesions was accelerated in *SDE5640*‐transgenic citrus compared with the wild type, implying enhanced multiplication of *Xcc* within the host tissues (Figure S1C–E). We further evaluated the proteasome activity and ubiquitination levels in *SDE5640*‐transgenic citrus. Transgenic lines exhibited substantially higher total ubiquitination levels and lower proteasome activity compared to WT plants (Figure 1D,E). Additionally, we extracted RNA from leaves infected with HLB to assess the expression of genes associated with SA‐mediated signal transduction. The results of RT‐qPCR revealed that the genes *CsNPR1*, *CsTGA*, *CsPR3*, and *CsPR5* were all significantly downregulated in the transgenic lines infected with HLB (Figure S1F–I). In addition, the SA content level was remarkably reduced in *SDE5640* transgenic citrus leaves compared with the WT control (Figure 1F). Overall, these results indicate that SDE5640 inhibits proteasome activity and promotes citrus susceptibility to *C*Las and *Xcc* infection.

**Figure 1:**
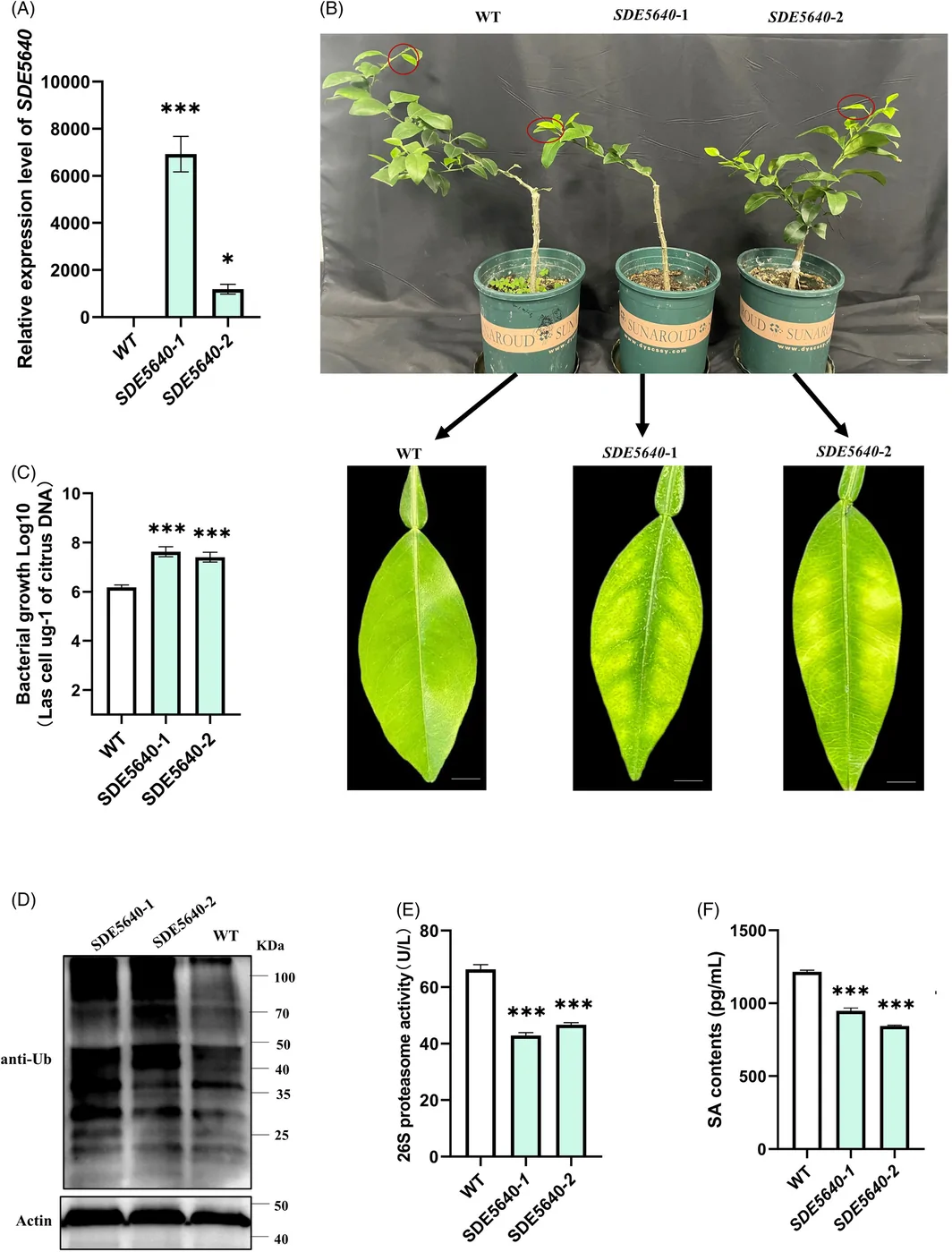
SDE5640 promotes bacterial proliferation in citrus by suppressing SA accumulation. (A) Reverse transcription quantitative PCR analysis of *SDE5640* in different transgenic lines. Error bars indicate standard errors of means, and the letters represent significant differences among samples by one‐way analysis of variance followed by LSD post hoc test （*P < 0.05; ***P < 0.001). (B) Leaf images taken at 6 month post‐inoculation (mpi) with *C*Las via budding and branch grafting. Healthy wild‐type (WT) and transgenic plants (pLGN‐SDE5640). (C) Quantitative analysis of *C*Las growth at 6 months after infection (MAI). The bacterial populations (*C*Las cells μg^−1^ of citrus DNA) were determined using qPCR. Standard errors were calculated from three plants per line. Error bars indicate standard errors of means, and the letters represent significant differences among samples by one‐way analysis of variance followed by LSD post hoc test (***P < 0.001). (D) Accumulation of total ubiquitinated proteins in leaves of *SDE5640* transgenic citrus plants compared with WT. (E) Proteasome activity in leaves of *SDE5640* transgenic citrus plants was determined; WT serves as the control. Error bars indicate standard errors of means, and the letters represent significant differences among samples by one‐way analysis of variance followed by LSD post hoc test (***P < 0.001). (F) SA content in *SDE5640* transgenic citrus plants. Error bars indicate standard errors of means, and the letters represent significant differences among samples by one‐way analysis of variance followed by LSD post hoc test (***P < 0.001).

### SDE5640 physically interacts with CsPSMD10 and CsPSMD10 can form dimers

To reveal the virulence function of SDE5640, an Immunoprecipitation‐Mass Spectrometry (IP‐MS) experiment was performed to identify its *in vivo* interactors (Table S1). The expression of *SDE5640* in *Nicotiana benthamiana* was detected via anti‐GFP immunoblotting. We further selected the *N. benthamiana* 26S proteasome regulatory subunit 10 (PSMD10) for subsequent analysis, given its well‐established roles in autophagy‐mediated immune modulation (Li et al., 2021; Luo et al., 2016). Sequence analysis revealed that *NbPSMD10* has homologs in the citrus genome (*CsPSMD10*/LOC102617699). A phylogenetic tree was constructed for PSMD10 homologs from *Malus domestica*, *Hibiscus syriacus*, *Neltuma alba*, *N. benthamiana*, *Cucumis sativus*, *Mangifera indica*, *Citrus clementina*, and *C. sinensis*. Among these species, *CsPSMD10* and *NbPSMD10* share a relatively close genetic relationship (Figure S2A). Using the Y2H system, the interaction between SDE5640 and CsPSMD10 was verified (Figure 2A). The SDE5640‐CsPSMD10 interaction was further confirmed by bimolecular fluorescence complementation (BiFC), coimmunoprecipitation (co‐IP) and pull‐down assays (Figure 2B–D). To identify the key regions involved in the interaction between SDE5640 and CsPSMD10, we constructed truncated variants of CsPSMD10 and performed a Y2H assay. The results demonstrated that SDE5640 interacts specifically with the conserved ankyrin repeat domains, Ank3 and Ank2, of PSMD10 (Figure S2B). In addition, SDE5640 was co‐localized with CsPSMD10 in the nucleus and cell membrane, and their interaction did not affect their subcellular location (Figure S2C). Together, these results demonstrate that the SDE5640 protein directly interacts with the CsPSMD10 protein both *in vivo* and *in vitro*.

**Figure 2:**
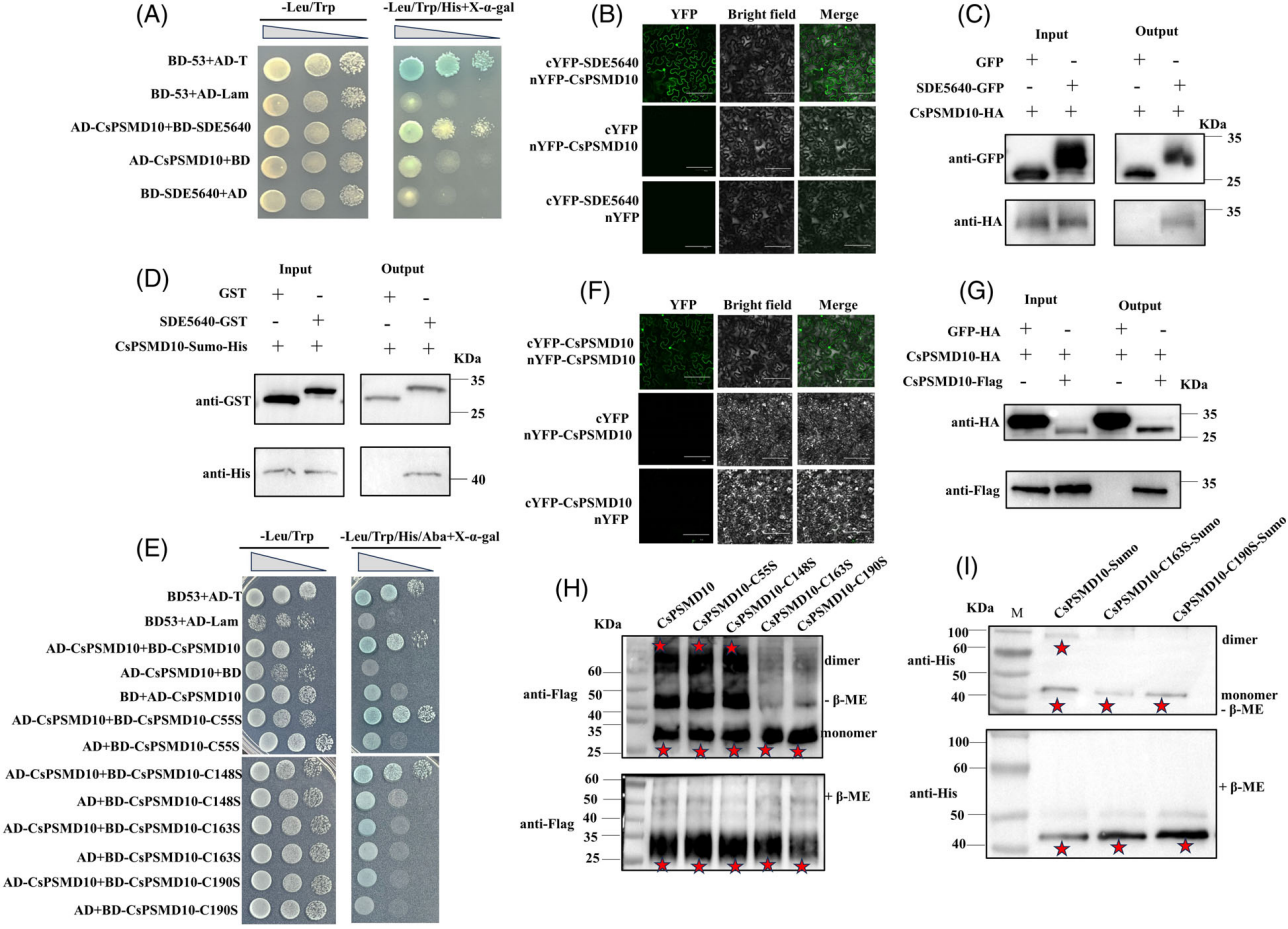
SDE5640 physically interacts with CsPSMD10 and promotes its dimerization. (A) Yeast two‐hybrid assay showed the interaction between SDE5640 and CsPSMD10 in yeast cells. BK‐53 and AD‐T served as positive control, BK‐53 and AD Lam served as negative control. (B) Bimolecular fluorescence complementation assay showed the interaction between SDE5640 and CsPSMD10 *in vivo*. Combinations of cYFP‐SDE5640 + nYFP‐CsPSMD10, nYFP‐SDE5640 +YFP, cYFP + nYFP‐CsPSMD10, were transiently co‐expressed in leaves of *Nicotiana benthamiana*. Scale bar = 100 μm. (C) Coimmunoprecipitation assay shows that SDE5640 interacts with CsPSMD10 *in vivo*. Input is the positive control group, representing the detection of the target protein in the extracted total protein, and output represents the protein detection after immunoprecipitation. (D) Pull‐down assay shows that SDE5640 interacts with CsPSMD10 *in vitro*. Input is the positive control group, representing the detection of the target protein in the extracted total protein, and output represents the protein detection after immunoprecipitation. (E) Yeast two‐hybrid assay showed the interaction between CsPSMD10 and CsPSMD10 in yeast cells, but replace all four cysteine residues with serine residues, respectively. The results show that CsPSMD10‐C55S and CsPSMD10‐C148S interact with themselves, but not with CsPSMD10‐C163S and CsPSMD10‐C190S. BK‐53 and AD‐T served as positive control, BK‐53 and AD Lam served as negative control. (F) Bimolecular fluorescence complementation assay showed the interaction between CsPSMD10 and CsPSMD10 *in vivo*. Combinations of cYFP‐CsPSMD10 + nYFP‐CsPSMD10, cYFP‐CsPSMD10 + nYFP, cYFP + nYFP‐CsPSMD10, were transiently co‐expressed in leaves of *N. benthamiana*. Scale bar = 100 μm. (G) Coimmunoprecipitation (Co‐IP) assay confirms that CsPSMD10 exhibits self‐interaction and forms homodimers in plant cells. Input indicates total protein lysates prior to immunoprecipitation, and IP represents immunoprecipitated protein complexes. (H) Detection of CsPSMD10 oligomerization *in vivo*. Total protein extracted from *N. benthamiana* leaves transiently expressing CsPSMD10‐Flag, CsPSMD10‐C55S‐Flag, CsPSMD10‐C148S‐Flag, CsPSMD10‐C163S‐Flag, and CsPSMD10‐C190S‐Flag were treated with or without 5% β‐ME and detected by western blotting with anti‐Flag antibody. (I) Detection of CsPSMD10 oligomerization *in vitro*. Total protein extracted from *Escherichia coli* expressing CsPSMD10: His, CsPSMD10‐C163S‐His, and CsPSMD10‐C190S‐His were treated with or without 5% β‐ME and detected by western blotting with anti‐His antibody.

PSMD10 homodimerization has been previously characterized in live mammalian cells (Luo et al., 2016). Here, Y2H and BiFC assays consistently demonstrated self‐interaction of CsPSMD10, indicating the capacity of CsPSMD10 to form homodimers (Figure 2E,F). Co‐IP assays further confirmed that homo‐interaction (Figure 2G). To identify the cysteine (C) residues responsible for CsPSMD10 homodimerization, we generated four single‐site mutants by individually substituting cysteine residues with serine (S) residues. Y2H assays showed that C55S and C148S mutants retained self‐interaction capability, while C163S and C190S mutants completely abolished CsPSMD10 homo‐interaction (Figure 2E).

We further transiently expressed wild‐type and mutant CsPSMD10 constructs in *N. benthamiana* leaves for immunoblotting analysis. Both dimeric and monomeric forms of CsPSMD10 were detected in leaf tissues expressing CsPSMD10, CsPSMD10‐C55S, and CsPSMD10‐C148S. In contrast, only the monomeric form was detected in leaves expressing CsPSMD10‐C163S and CsPSMD10‐C190S (Figure 2H). Consistently, only the monomeric CsPSMD10 was observed *in vitro* expressing CsPSMD10‐C163S and CsPSMD10‐C190S (Figure 2I). Moreover, Y2H verification revealed that SDE5640 interacts with both the monomer and dimer of CsPSMD10 (Figure S2D). Collectively, these findings demonstrate that the disulfide bonds mediated by Cys163 and Cys190 are essential for maintaining the stability of the PSMD10 homodimers.

### CsPSMD10 negatively regulates citrus immunity

Next, we investigated the tissue‐specific expression pattern of CsPSMD10 in citrus by reverse transcription quantitative real‐time PCR (RT‐qPCR). The results showed that CsPSMD10 was expressed in leaf and stem tested, with the highest expression in leaf veins, and relatively low expression in stem mesophyll tissues (Figure S3A). In addition, the transcription level of *CsPSMD10* is significantly downregulated under HLB infection (Figure S3B). To elucidate the potential roles of CsPSMD10 in plant immunity, five independent *CsPSMD10*‐expressing citrus lines were obtained and two of them (*CsPSMD10*‐1 and *CsPSMD10*‐2) were selected for subsequent studies (Figure S3C,D). We performed graft‐inoculated using *C*Las‐infected buds on *CsPSMD10*‐transgenic citrus lines and WT controls and confirmed successfully pathogen transmission. *C*Las titers quantification showed that the transgenic plants were more susceptible to *C*Las (Figure 3A–C). We next generated transgenic citrus hairy roots overexpressing *CsPSMD10* and the *CsPSMD10‐*C190S mutant; positive transgenic hairy root lines were verified by PCR amplification (Figure S3E). Subsequent HLB resistance assays revealed that citrus hairy roots expressing the *CsPSMD10‐C190S* mutant exhibited markedly improved resistance against HLB (Figure 3D–F).

**Figure 3:**
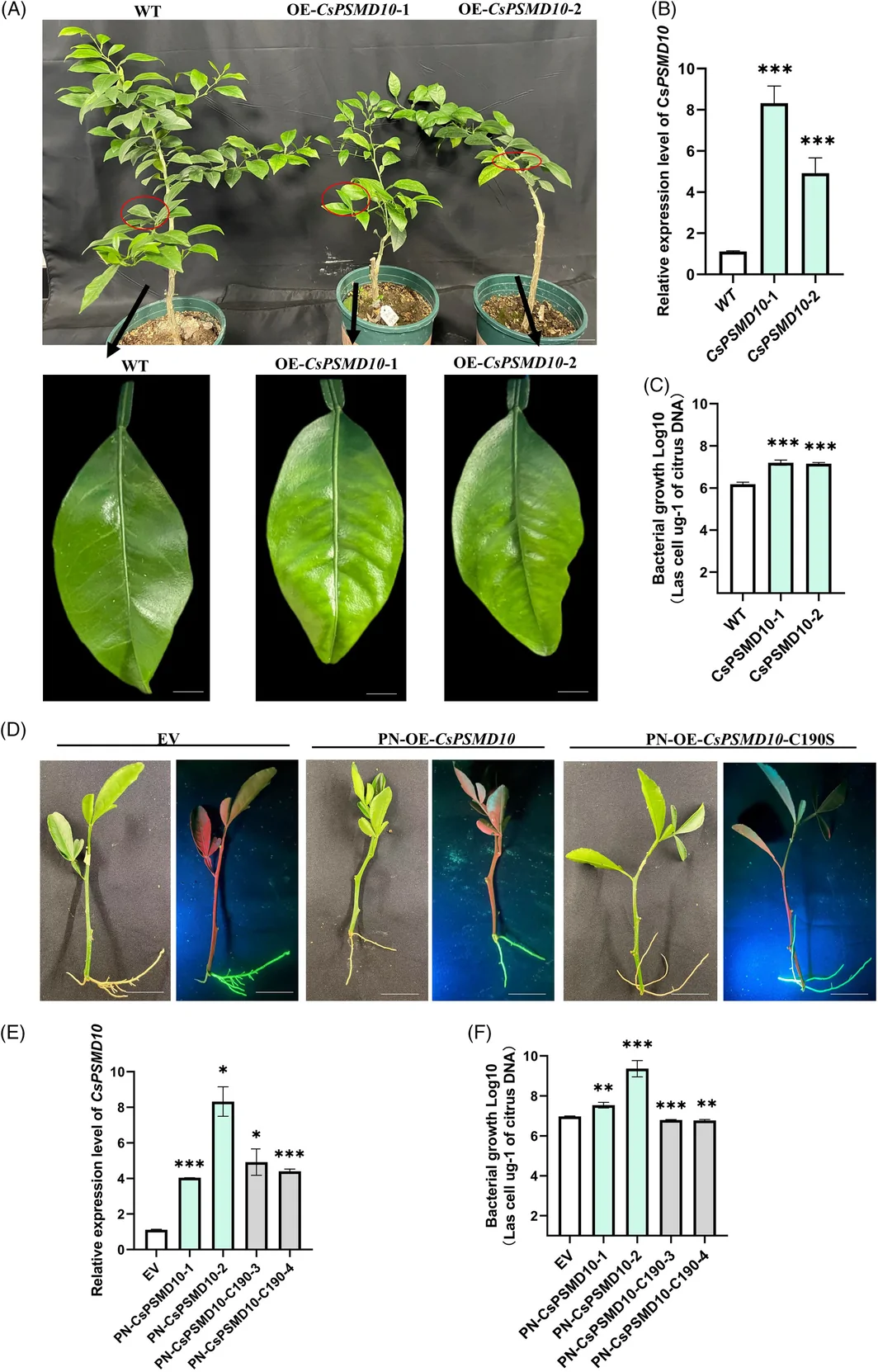
CsPSMD10 negatively regulates citrus immunity. (A) Leaf images taken at 6 month post‐inoculation (mpi) with *C*Las via budding and branch grafting. Healthy wild‐type (WT) and transgenic plants (pLGN‐CsPSMD10). (B) Reverse transcription quantitative PCR analysis of *CsPSMD10* in different transgenic lines. Error bars indicate standard errors of means, and the letters represent significant differences among samples by one‐way analysis of variance followed by LSD post hoc test (***P < 0.001). (C) Quantitative analysis of *C*Las growth at 6 months after infection (MAI). The bacterial populations (*C*Las cells μg^−1^ of citrus DNA) were determined using qPCR. Standard errors were calculated from three plants per line. Error bars indicate standard errors of means, and the letters represent significant differences among samples by one‐way analysis of variance followed by LSD post hoc test (***P < 0.001). (D) Green fluorescence signal visualized by the fluorescence signal with a portable excitation lamp in transgenic tissues of citrus roots. Scale bars: 2 cm. (E) The relative expression levels of *SDE4250* in transgenic plants were determined by normalizing against its expression in the WT control, using the citrus β‐actin gene as an internal reference. Error bars indicate standard errors of means, and the letters represent significant differences among samples by one‐way analysis of variance followed by LSD post hoc test (*P < 0.05; ***P < 0.001). (F) Quantitative analysis was conducted to assess the growth of *C*Las during a 20 days period following *Agrobacterium* induction in CsPSMD10 transgenic citrus and CsPSMD10‐C190S plants. CsPSMD10 expression and WT plants were each subjected to three independent biological replicates. The bacterial populations (*C*Las cells μg^−1^ of citrus DNA) were quantified using qPCR. Error bars indicate standard errors of means, and the letters represent significant differences among samples by one‐way analysis of variance followed by LSD post hoc test (**P < 0.01; ***P < 0.001).

To further validate the function of *CsPSMD10*, we employed a citrus leaf blotch virus (CLBV) vector to silence this gene in citrus. qRT‐PCR confirmed effective *CsPSMD10* silencing, with no detectable impacts on plant growth and development (Figure S3F,G). As expected, *CsPSMD10*‐silenced plants displayed stronger resistance to *Xcc* than control plants. Notably, SDE5640 expression could no longer promote *Xcc* proliferation in *CsPSMD10*‐silenced leaves when compared with GUS‐silenced leaves (Figure S3H–J). Collectively, these results suggest that SDE5640 inhibits citrus innate immunity likely in a *CsPSMD10‐*dependent manner.

We further performed RT‐qPCR analysis to examine the expression of genes involved in the SA‐mediated signaling pathway in *CsPSMD10* transgenic citrus. As observed, the expression of *CsNPR1, CsTGA, CsPR3*, and *CsPR5* was substantially suppressed in these transgenic plants (Figure S4A–D). Correspondingly, we detected a notable reduction in endogenous SA content in transgenic citrus (Figure S4E).

### SDE5640 promotes CsPSMD10‐dependent autophagy in plants

Since PSMD10 has been reported to regulate autophagy‐mediated immunity in mammals (Luo et al., 2016), we investigated whether CsPSMD10 modulates autophagy in plants. To visualize cytoplasmic autophagosomes and vacuolar autophagic bodies, we used GFP‐tagged NbATG8f (GFP‐NbATG8f) as a reporter. Compared with GUS‐Flag control plants, *N. benthamiana* transiently expressing Flag‐CsPSMD10 displayed markedly fewer GFP‐labeled autophagic bodies, with autophagic activity reduced by two–threefold (Figure S5A–C). TEM analysis showed that after 12 h of E‐64d treatment, *CsPSMD10*‐overexpressing citrus lines had fewer autophagosomes than WT plants (Figure 4A,B). NBR1 acts as a selective autophagy cargo receptor, and its accumulation in the plant vacuole increases when autophagy is inhibited. We then examined NBR1 protein abundance in WT and two independent *CsPSMD10*‐transgenic lines. Western blot analysis confirmed greater NBR1 accumulation in the two transgenic lines relative to WT (Figure 4C). Taken together, these results demonstrate that CsPSMD10 suppresses autophagy activity in transgenic citrus plants.

**Figure 4:**
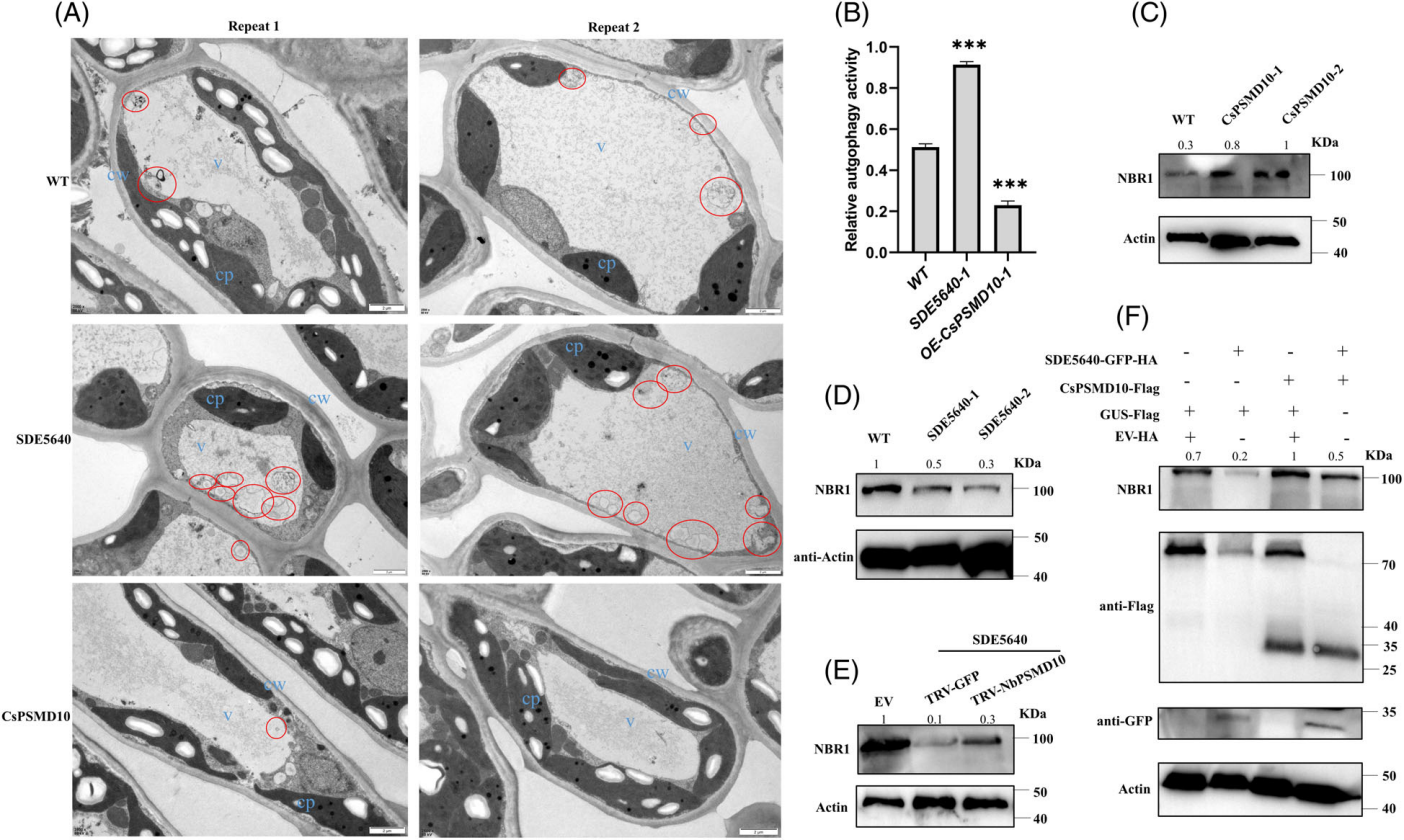
SDE5640 promotes CsPSMD10‐dependent autophagy in plants. (A) Representative TEM images of autophagic bodies in *SDE5640*‐transgenic citrus plants, *CsPSMD10*‐transgenic citrus and WT plants. Red arrowheads and circles indicate the autophagic bodies inside the vacuole. The red boxes indicate areas which are enlarged and shown on the right side panels. Cp, chloroplast; CW, cell wall; M, mitochondria; V, vacuole. Scale bars = 2 μm (left) and 1 μm (right). (B) Relative autophagic activity in SDE5640 plants and CsPSMD10 plants were normalized to that of WT plants. Approximately 50 cells were quantified in each treatment. Values represent means ± SE from three independent experiments. Asterisks indicate significant differences between the transgenic citrus (OE lines) and the WT plants by one‐way ANOVA followed by LSD post hoc test (***P < 0.001). (C, D) Immunoblot analysis of NBR1 protein expression in transgenic citrus (OE lines) and WT plants. Actin was used as a loading control in western blot analysis. The experiment was repeated at least three times with similar results. (E) Immunoblot analysis of NBR1 protein expression 2 days after transient expression of SDE5640 and*CsPSMD10* in *Nicotiana benthamiana*. Actin was used as a loading control in western blot analysis. The experiment was repeated at least three times with similar results. (F) Immunoblot analysis of NBR1 protein expression 2 days after transient expression of SDE5640 in TRV‐*NbPSMD10*. Actin was used as a loading control in western blot analysis. The experiment was repeated at least three times with similar results.

Given the SDE5640‐CsPSMD10 physical interaction and the regulation function of CsPSMD10 in autophagy, we next explored the direct effect of SDE5640 on plant autophagy. Transient expression of GFP‐tagged SDE5640 in *N. benthamiana* induced abundant autophagic body formation, with autophagic level increased two–threefold relative to the GUS‐Flag control (Figure S6A–C). A conserved pro‐autophagic effect was observed in citrus: transgenic citrus leaves overexpressing *SDE5640* displayed substantially more autophagic bodies and reduced NBR1 protein accumulation compared with wild‐type controls (Figure 4D).

Considering the opposing roles of CsPSMD10 and SDE5640 in modulating autophagy, we further examined whether SDE5640‐triggered autophagy relies on CsPSMD10 function. Upon *NbPSMD10* silencing in *N. benthamiana*, the pro‐autophagic capacity of transiently expressed *SDE5640* was markedly compromised, as evidenced by restored NBR1 accumulation (Figure 4E). Furthermore, co‐expression of *SDE5640* with *CsPSMD10* confirmed that SDE5640 could efficiently alleviate CsPSMD10‐mediated autophagic suppression (Figure 4F). Collectively, these results demonstrate that SDE5640 activates plant autophagy in a CsPSMD10‐dependent manner.

### CsPSMD10 mediates CsATG8c degradation through the 26S proteasome system

Previous work has shown that PSMD10 induces autophagy and promotes tumor progression by interacting with ATG7 (Luo et al., 2016). Given the regulatory role of the SDE5640‐CsPSMD10 interaction complex in citrus autophagy, we examined whether CsPSMD10 associates with CsATG7 proteins. Y2H assays detected no interaction between CsPSMD10 and CsATG7 (Figure S7A). In contrast, CsPSMD10 was found to interact with CsATG8 family proteins (Figure 5A). The *in vivo* interaction between CsATG8c and CsPSMD10 was confirmed via BiFC in *N. benthamiana* (Figure 5B). Co‐IP assays further validated the physical interactions of CsPSMD10 with CsATG8c, CsATG8d, and CsATG8f (Figure 5C). Together, these results demonstrate that the CsPSMD10 directly interacts with the CsATG8, uncovering a plant‐specific interaction pattern distinct from the mammalian PSMD10‐ATG7 regulatory module.

**Figure 5:**
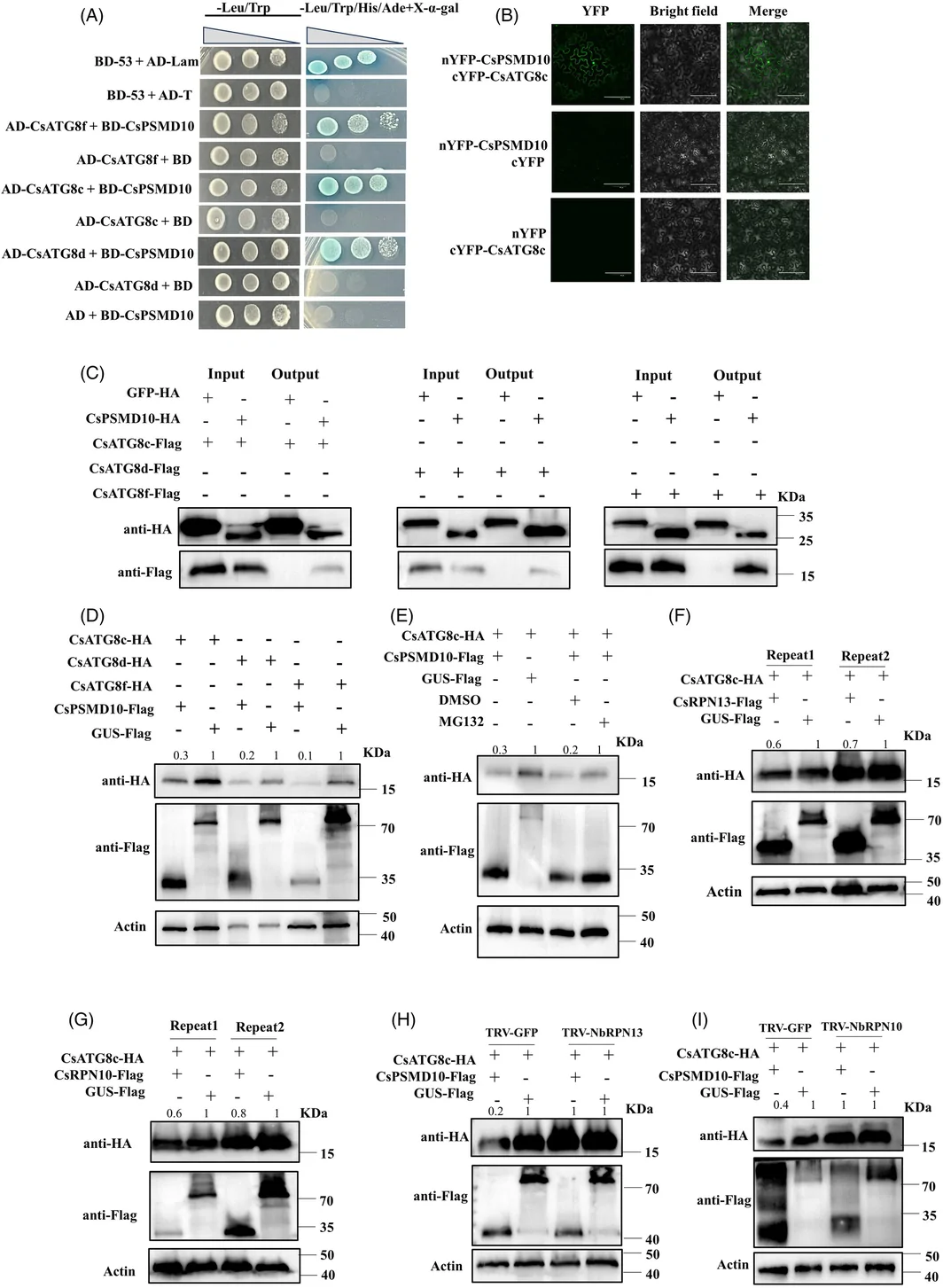
CsPSMD10 mediates CsATG8c degradation through the 26S proteasome system. (A) Yeast two‐hybrid assay showed the interaction between CsPSMD10 and CsATG8s in yeast cells. BK‐53 and AD‐T served as positive control, BK‐53 and AD Lam served as negative control. (B) Bimolecular fluorescence complementation assay showed the interaction between CsPSMD10 and CsATG8c *in vivo*. Combinations of cYFP‐CsATG8c + nYFP‐CsPSMD10, cYFP‐CsATG8c + nYFP, cYFP + nYFP‐CsPSMD10, were transiently co‐expressed in leaves of *Nicotiana benthamiana*. Scale bar = 100 μm. (C) Coimmunoprecipitation assay shows that CsPSMD10 interacts with CsATG8c, CsATG8d and CsATG8f *in vivo*. Input is the positive control group, representing the detection of the target protein in the extracted total protein, and output represents the protein detection after immunoprecipitation. (D) Immunoblot analysis of CsATG8s protein degradation in *N. benthamiana* leaves inoculated with CsATG8s‐HA + CsPSMD10‐Flag, CsATG8s‐HA + GUS‐Flag, Protein degradation was examined by western blot analysis using specific antibodies, actin was used as a loading control in Immunoblot analysis. The quantification was calculated according to the software ImageJ. (E) CsATG8c‐HA together with CsPSMD10‐FLAG were transiently co‐expressed in *N. benthamiana* leaves via agroinfiltration, and the infiltrated leaves were treated with DMSO and MG132 at 24 hpi and examined at 48 dpi. Protein accumulation was examined by Immunoblot analysis using specific antibodies. Actin was used as a loading control in western blot analysis. The quantification was calculated according to the software ImageJ. (F, G) Immunoblot analysis of CsATG8c protein degradation in *N. benthamiana* leaves inoculated with CsATG8c‐HA + CsRPN13‐Flag/CsRPN10‐Flag, CsATG8c‐HA + GUS‐Flag, Protein degradation was examined by western blot analysis using specific antibodies, actin was used as a loading control in Immunoblot analysis. (H, I) Silencing of *NbRPN10* and *NbRPN13* in *N. benthamiana* at 10 days after agroinfiltration with CsATG8c‐HA and CsPSMD10‐FLAG. Western blot analysis of CsATG8c‐HA protein accumulation in *N. benthamiana* leaves. Actin was used as a loading control in western blot analysis.

PSMD10 is a non‐ATPase subunit of the 26S proteasome and acts as a molecular chaperone that binds target proteins to facilitate their proteasomal degradation (Dawson et al., 2002). To further elucidate the functional effect of the CsPSMD10‐CsATG8s interaction on autophagy. we transiently expressed CsATG8 family proteins in *N. benthamiana* leaves. Western blotting assays showed that CsPSMD10 induced the degradation of CsATG8c, CsATG8d, and CsATG8f (Figure 5D). Moreover, transcript levels of *CsATG8* family genes were significantly reduced in *CsPSMD10* transgenic plants (Figure S7B–D).

To explore the mechanism underlying CsPSMD10‐mediated CsATG8c degradation, we co‐expressed CsATG8c with CsPSMD10 or GUS‐Flag in *N. benthamiana* leaves and treated the infiltrated leaves with different protease inhibitors. Pharmacological inhibition of the 26S proteasome by MG132 substantially rescued CsATG8c from CsPSMD10‐induced degradation (Figure 5E). The 26S proteasome consists of two core subcomplexes: the 20S core particle (CP) and the 19S regulatory particle (RP). As key RP subunits, RPN10 and RPN13 serve as ubiquitin receptors that recruit ubiquitinated substrates for proteasomal degradation (Hamazaki et al., 2015). We transiently co‐expressed CsATG8c with GUS‐Flag, RPN10‐Flag, or RPN13‐Flag in *N. benthamiana* leaves. Immunoblot assays showed that both RPN10 and RPN13 markedly reduced CsATG8c protein abundance (Figure 5F,G). In line with these results, significantly higher accumulation of endogenous CsATG8c was detected in *NbRPN10*‐ and *NbRPN13*‐silenced plants relative to the TRV‐GFP control lines (Figure 5H,I).

### SDE5640 inhibits PSMD10‐ATG8c interaction

Our previous results have confirmed that the SDE5640‐CsPSMD10 interaction activates citrus autophagy, whereas CsPSMD10 directly targets and degrades CsATG8c to suppress autophagy. We therefore hypothesized that SDE5640 may competitively interfere with the CsPSMD10‐CsATG8c interaction to alleviate the inhibitory effect of CsPSMD10 on autophagy. To validate this hypothesis, we performed BiFC assays to assess whether SDE5640 modulates PSMD10‐ATG8c association. *N. benthamiana* leaves were co‐infiltrated with CsPSMD10‐nYFP and CsATG8c‐cYFP, together with either CFP‐SDE5640 or CFP control. Fluorescence intensity was substantially attenuated in the CFP‐SDE5640‐expressing group compared with the CFP group (Figure 6A,B). A competitive Co‐IP assay further verified this regulatory effect. CsATG8c‐HA and CsPSMD10‐Flag were co‐expressed with HA‐SDE5640 or HA‐GUS in *N. benthamiana* leaves. Compared with the control, HA‐SDE5640 markedly weakened the binding between CsPSMD10‐Flag and CsATG8c‐HA, leading to reduced coimmunoprecipitation efficiency (Figure 6C). Moreover, *in vitro* competitive pull‐down assays confirmed that SDE5640 disrupts the CsPSMD10‐CsATG8c interaction in a dose‐dependent manner (Figure 6D). Collectively, these findings indicate that SDE5640 competitively binds CsPSMD10 to block its association with CsATG8c.

**Figure 6:**
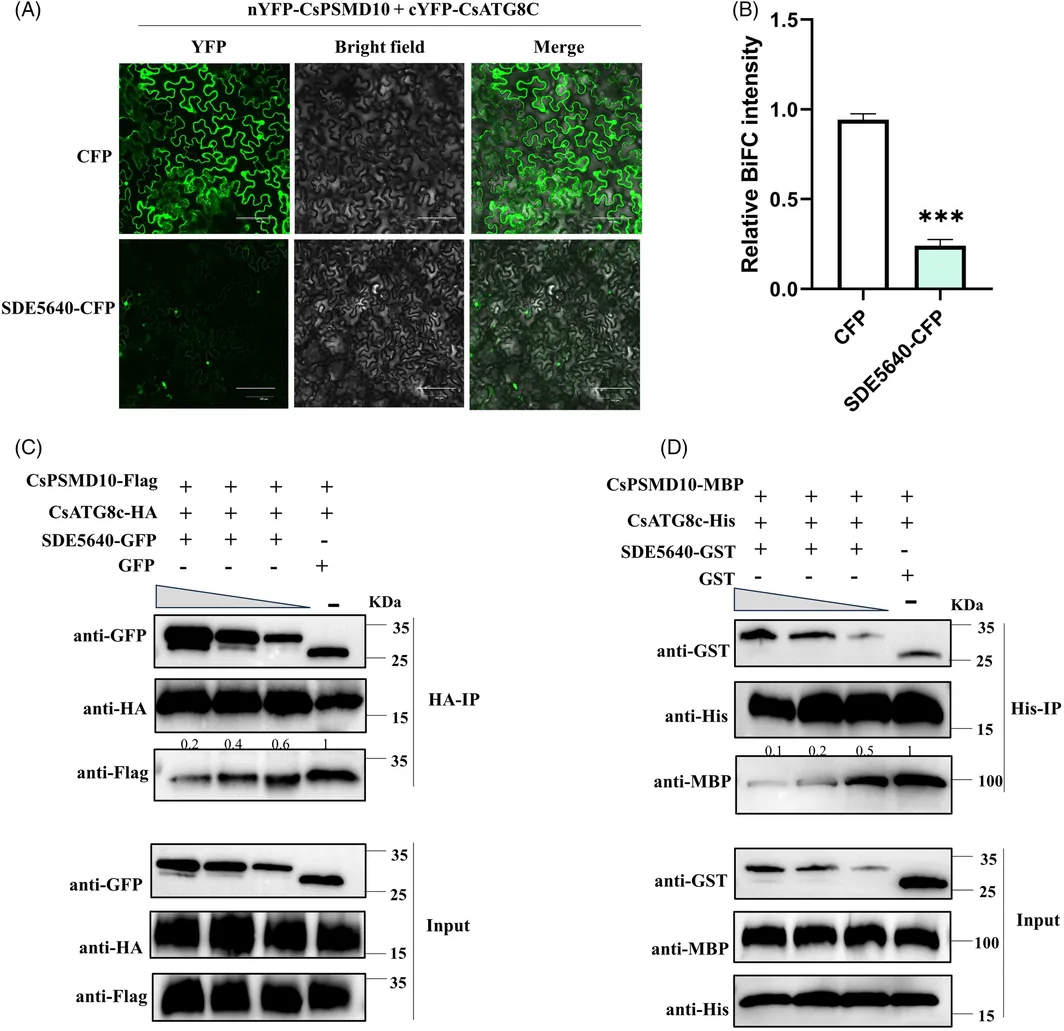
SDE5640 activates autophagy by inhibiting PSMD10 homodimer with ATG8c. (A, B) Bimolecular fluorescence complementation assay showed that SDE5640 suppressed CsPSMD10 and CsATG8c interaction. Error bars indicate standard errors of means, and the letters represent significant differences among samples by one‐way analysis of variance followed by LSD post hoc test (***P < 0.001). (C) Co‐IP assays showing the influence of SDE5640 on the interaction between CsPSMD10 and CsATG8c. CsPSMD10‐Flag and CsATG8c‐HA with increasing amounts of SDE5640 were co‐infiltrated into *Nicotiana benthamiana* leaves. At 48 hpi, total proteins were extracted and incubated with HA magnetic agarose beads. Western blotting assays analyzed input and IP proteins with anti‐GFP, anti‐Flag, or anti‐HA antibodies. (D) Competitive His pull‐down assays *in vitro*. CsPSMD10‐MBP, CsPSMD10‐His, and GST‐SDE5640 were purified individually from *Escherichia coli*. The increasing amounts of GST‐SDE5640 were incubated with CsPSMD10‐MBP and CsPSMD10‐His together. Western blotting assays detected input and pull‐down proteins with an anti‐MBP, an anti‐GST, or an anti‐His antibody.

### SDE5640 induces autophagy to promote PBA1 degradation

Autophagy mediates proteasome turnover under nutrient deficiency and proteasome inhibition conditions, a process termed proteaphagy (Marshall et al., 2015). In *Arabidopsis*, RPN10 acts as a selective autophagy receptor that targets inactive 26S proteasomes via dual interactions with ubiquitinated proteasome subunits and membrane‐conjugated ATG8, thereby facilitating proteasome degradation through autophagic pathways (Marshall et al., 2015). Given that SDE5640 suppresses proteasome activity (Mei et al., 2025) and activates citrus autophagy, we further explored whether SDE5640 induces proteaphagy in citrus. Consistent with the effects of the proteasome inhibitor MG132, SDE5640 expression significantly elevated the protein abundance of citrus 26S proteasome subunits RPN10, RPN13, and RPT3 (Figure 7A,B).

**Figure 7:**
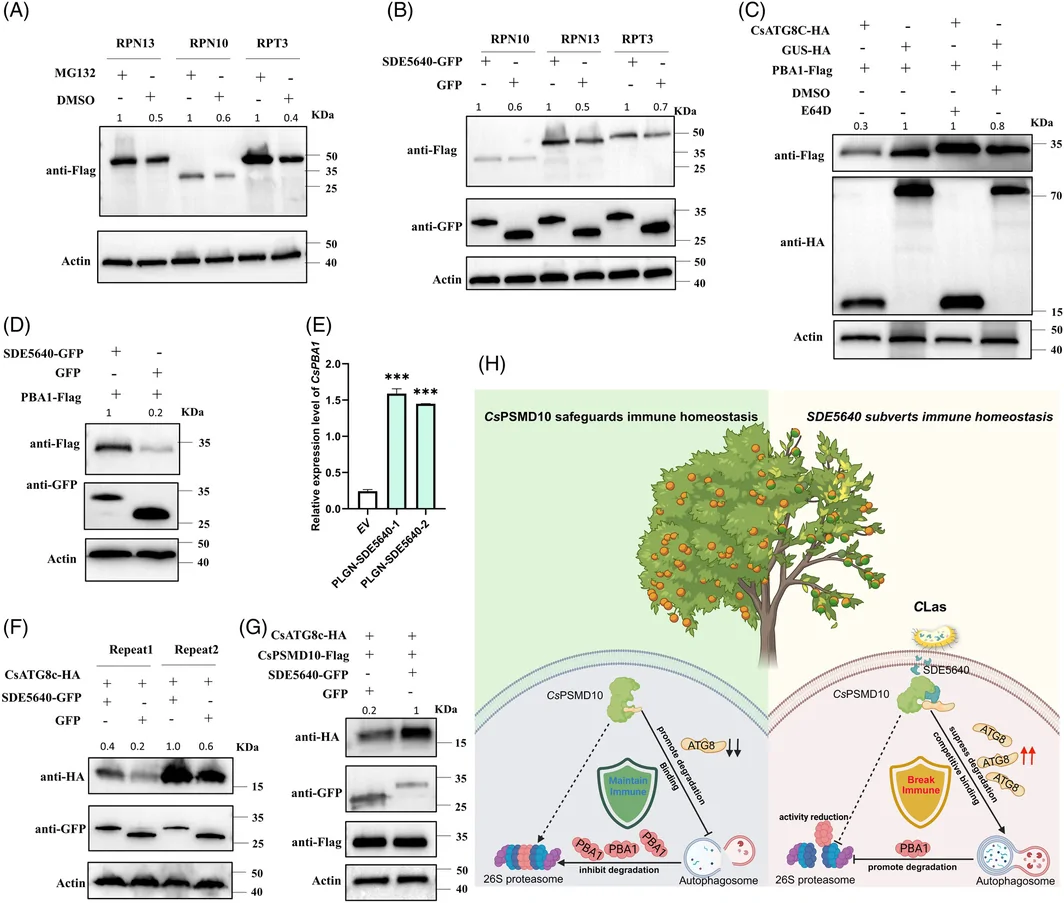
SDE5640 induces autophagy to promote PBA1 degradation. (A) Immunoblot analysis of proteasome subunit protein accumulation in *Nicotiana benthamiana* leaves inoculated with MG132, Protein accumulation was examined by western blot analysis using specific antibodies, actin was used as a loading control in Immunoblot analysis. (B) Immunoblot analysis of proteasome subunit protein accumulation in *N. benthamiana* leaves inoculated with SDE5640‐GFP + CsRPN10/RPN13/RPT3‐Flag, GFP + CsRPN10/RPN13/RPT3‐Flag, Protein accumulation was examined by western blot analysis using specific antibodies, actin was used as a loading control in Immunoblot analysis. (C) Immunoblot analysis of CsPBA1 protein in *N. benthamiana* leaves inoculated with CsPBA1‐Flag + CsATG8c‐HA, CsPBA1‐Flag + GUS‐HA, Protein was examined by western blot analysis using specific antibodies, actin was used as a loading control in Immunoblot analysis. (D) Immunoblot analysis of CsPBA1 protein in *N. benthamiana* leaves inoculated with CsPBA1‐Flag + SDE5640‐GFP, CsPBA1‐Flag + GFP, Protein was examined by western blot analysis using specific antibodies, actin was used as a loading control in Immunoblot analysis. (E) Reverse transcription quantitative PCR analysis of *PBA1* in *SDE5640* transgenic lines. Error bars indicate standard errors of means, and the letters represent significant differences among samples by one‐way analysis of variance followed by LSD post hoc test (***P < 0.001). (F) Immunoblot analysis of CsATG8c protein in *N. benthamiana* leaves inoculated with SDE5640‐GFP + CsATG8c‐HA, GFP + CsATG8c‐HA, Protein was examined by western blot analysis using specific antibodies, actin was used as a loading control in Immunoblot analysis. (G) SDE5640 can enhance the accumulation of ATG8C protein and inhibit the degradation of CsATG8c by CsPSMD10. (H) In healthy citrus cells, CsPSMD10 directly binds CsATG8 and targets it for ubiquitin‐dependent degradation by the intact 26S proteasome, maintaining low free CsATG8 levels and suppressing autophagy to preserve immune homeostasis. Upon *Candidatus* Liberibacter infection, the pathogen effector SDE5640 competitively disrupts the CsPSMD10–CsATG8c interaction, allowing CsATG8c to escape proteasomal degradation and accumulate. The resulting high CsATG8c levels induce autophagy, which mediates degradation of the 20S proteasome core subunit CsPBA1, leading to 26S proteasome disassembly, accumulation of ubiquitinated proteins, and ultimately host immune suppression.

MG132, a peptide aldehyde compound, covalently binds to the active site of the β‐subunit (PBA) of the 20S proteasome to specifically block 20S catalytic activity, thereby disabling the proteolytic function of the 26S proteasome complex (Gladman et al., 2016). We next examined the regulatory relationship between ATG8c and the 20S core subunit PBA1. Our results showed that CsATG8c promotes the degradation of PBA1, and treatment with the autophagy inhibitor E‐64d restored the protein level of PBA1 (Figure 7C). Notably, no physical interaction was detected between CsATG8c and PBA1 (Figure S7E), indicating that CsATG8c‐mediated PBA1 degradation occurs in an indirect manner.

Intriguingly, SDE5640 markedly induced both the transcript and protein levels of PBA1 (Figure 7D,E), suggesting that SDE5640 triggered the transcriptional upregulation of proteasome subunits masks their autophagic degradation, resulting in net accumulation of proteasome components. Further assays revealed that SDE5640 enhances the CsATG8c protein accumulation and efficiently blocks CsPSMD10‐mediated CsATG8c degradation (Figure 7F,G). Collectively, these results demonstrate that SDE5640 impairs proteasome function by stabilizing CsATG8c, which in turn promotes autophagy‐dependent degradation of the 20S proteasome subunit PBA1 in citrus.

## DISCUSSION

The ubiquitin‐26S proteasome system (UPS) and autophagy constitute two conserved, core protein degradation cascades governing plant innate immunity, and phytopathogens deploy secreted effector proteins to perturb their crosstalk for successful colonization. Multiple *C*Las effectors are known to individually manipulate autophagy or UPS, yet how a single effector simultaneously rewires both pathways remains poorly defined. Here, we characterized the virulence effector SDE5640 and uncovered a novel regulatory axis SDE5640‐CsPSMD10‐CsATG8c‐CsPBA1 that simultaneously suppress proteasome activity and boosts pathogenic autophagy, advancing mechanistic understanding of *C*Las immune subversion (Figure 7H).

PSMD10 (gankyrin) acts as a well‐documented autophagy promoter in mammalian cells via direct interaction with ATG7 (Luo et al., 2016), whereas its physiological roles in plants remain largely uncharacterized before the present work. Multiple biochemical and genetic lines of evidence in this study uncover a plant‐unique regulatory mechanism of citrus CsPSMD10 that differs sharply from its animal orthologs. Unlike mammalian PSMD10 which binds ATG7 to activate autophagy, citrus CsPSMD10 undergoes homodimerization relying on disulfide bonds formed by Cys163 and Cys190 residues; it directly targets multiple CsATG8 isoforms and mediates their degradation via the 26S proteasome, thus persistently restraining autophagic flux. In line with its autophagy‐suppressive function, overexpression of CsPSMD10 weakens salicylic acid (SA)‐mediated immune signaling and renders citrus more susceptible to *C*Las and *Xcc* infections. In contrast, the dimerization‐deficient mutant CsPSMD10‐C190S significantly enhances citrus resistance against HLB in hairy root functional assays, demonstrating that homodimerization of CsPSMD10 is an indispensable prerequisite for its negative regulatory role in plant immunity.

Notably, SDE5640 exhibits physical interaction with CsPSMD10 *in planta*. While our biochemical data confirm that CsPSMD10 forms functional homodimers to mediate its physiological functions, the precise regulatory circuit linking SDE5640 and CsPSMD10 homodimerization, as well as how SDE5640 rewires the dimer‐mediated turnover cascade of CsATG8c, remain incompletely resolved by our present experimental findings. We aim to systematically dissect this molecular axis bridging SDE5640 and dimeric CsPSMD10 in subsequent mechanistic studies.

*C*Las encodes effectors with diverse effects on citrus autophagy: SDE3 accelerates ATG8 degradation to block autophagy, whereas SDE4405 directly binds ATG8 to trigger autophagic flux (Shi, Gong, et al., 2023; Shi, Yang, et al., 2023). Unlike these two effectors that target autophagy core components, SDE5640 adopts an indirect competitive binding strategy to relieve CsPSMD1‐mediated autophagy suppression. SDE5640 specifically interacts with the Ank2/Ank3 domains of CsPSMD10, dose‐dependently disrupting the CsPSMD10–CsATG8c complex and stabilizing CsATG8c protein. Genetic epistasis assays further validated that SDE5640‐induced autophagy is dependent on host CsPSMD10, establishing a unique upstream regulatory node among known *C*Las virulence proteins. Functionally, transgenic citrus expressing SDE5640 exhibited reduced proteasome activity, attenuated SA biosynthesis and repressed SA marker gene expression, collectively creating an immune‐compromised state favorable to pathogen proliferation.

Autophagic turnover of functional or damaged 26S proteasomes (termed proteaphagy) is a conserved stress response in eukaryotes. In Arabidopsis, RPN10 serves as a universal cargo receptor mediating autophagic clearance of intact proteasome holoenzymes upon proteasome inhibition (Marshall et al., 2015). The bacterial effector HopM1 from *P. syringae* hijacks this canonical proteaphagy pathway to suppress host proteolytic capacity (Üstün et al., 2018). Here, we identified a distinct proteasome‐targeting autophagy mode triggered by *C*Las: instead of targeting fully assembled proteasomes, SDE5640‐stabilized CsATG8c drives selective autophagic degradation of dissociated free PBA1, the catalytic core subunit of the 20S particle. No direct physical interaction was detected between CsATG8c and CsPBA1, implying unidentified bridging adaptor proteins facilitate this selective turnover. Loss of cellular CsPBA1 pools substantially impairs total proteasome activity, providing a molecular basis for SDE5640‐mediated immune suppression. SDE5640 elevates CsPBA1 transcript abundance while simultaneously promoting autophagic depletion of total CsPBA1 protein, resulting in a net reduction of functional proteasome machinery. Nevertheless, whether SDE5640 initiates canonical proteaphagy targeting intact 26S proteasome complexes awaits further experimental validation.

In summary, this study delineates a previously uncharacterized virulence cascade by which the *C*Las effector SDE5640 competitively sequesters CsPSMD10 to stabilize CsATG8c, induce selective autophagic degradation of the proteasome catalytic subunit CsPBA1, and ultimately dampen SA‐dependent citrus immunity. Our findings expand the repertoire of effector‐mediated UPS‐autophagy crosstalk manipulation in woody perennial pathosystems, uncover a plant‐specific immune regulatory function of CsPSMD10, and provide feasible molecular targets for genetic improvement of HLB resistance.

## EXPERIMENTAL PROCEDURES

### RT‐qPCR

The total RNA of midribs of sweet orange and *N. benthamiana* leaves was extracted using TRIzol Plus (Tiangen). DNA removal and cDNA synthesis were conducted using a PrimeScript RT Reagent Kit with gDNA Eraser (Takara) according to the manufacturer's instructions. The qPCR assays were conducted using the specific primers in Table S2 and the NovoStart SYBR Qpcr SuperMix Plus Kit (Novoprotein, E096‐01A) on a qTower3G real‐time PCR system.

### Generation of *SDE5640*and *CsPSMD10* ‐transgenic citrus

A plant binary expression vector carrying the cauliflower mosaic virus 35S (CaMV 35S) promoter was used to generate transgenic ‘Wanjincheng’ orange plants expressing SDE5640. The ‘Wanjincheng’ orange seeds collected from the National Citrus Germplasm Bank in Chongqing, China, were removed from the fruit and placed on solid Murashige and Skoog (MS) media on a clean bench. Transgenic plants were generated from the epicotyl explants of ‘Wanjincheng’ orange by agrobacterium‐mediated transformation as described above (Shi, Yang, et al., 2023). Gather the shoots and cut them into small pieces for infiltration of the GUS staining solution. A blue positive signal can be seen after incubation. Leave at 37°C for 2–4 hours. Subsequently, the leaves were collected for RT‐qPCR analysis.

### Y2H assays

The proteins for verifying the interaction were, respectively, constructed into the two vectors, pGBKT7 and pGADT7. The fragment insertion sites of the two vectors were both *Eco*RI and *Bam*HI. Two reorganization carriers were co‐transformed with a sweet orange plasmid library into *Saccharomyces cerevisiae* Y2H Gold cells (OE BioTech). Yeast transformants were selected on SD/−Leu/−Trp/−His/−Ade medium (Coolaber, YM2001‐1Set) supplemented with X‐α‐gal and aureobasidin A (AbA). The negative control pair was pGADT7‐T and pGBKT7‐lam, and the positive control pair was pGADT7‐T and pGBKT7‐p53. Transformants were cultured on a basic medium devoid of leucine and tryptophan for 4 days at 30°C. Subsequently, the yeast clones were transferred to a more stringently selective medium that lacked leucine, tryptophan, adenine, and histidine for approximately three to 6 days.

### BiFC assay

SDE5640 was inserted using the primer Table S2 and cloned into the *Bam*HI and *Sa1*I sites of pCV‐cYFP containing the C‐terminal fragment of YFP to generate SDE5640‐cYFP. The cDNA of CsPSMD10 and CsATG8c was cloned into the *Bam*HI and *Sa1*I sites of pCV‐nYFP containing the N‐terminal end of YFP to generate pCV‐nYFP‐CsPSMD10 or pCV‐nYFP‐CsATG8c (Du et al., 2023). At 48 hpi, the fluorescence signal was observed using a scanning confocal microscope (FV3000; Olympus). Each experiment was repeated at least three times.

### CLBV‐mediated virus‐induced gene silencing in citrus

A approximately 300 bp specific fragment of *CsPSMD10* was amplified by PCR. The amplified fragment was inserted into the CLBV viral vector to construct the recombinant vector CLBV‐CsPSMD10. The resulting recombinant plasmid was transformed into *A. tumefaciens* strain EHA105 (Table S3). The agrobacterial strains carrying CLBV‐CsPSMD10 and the empty control vector CLBV‐GUS‐HA were cultured and resuspended in infiltration buffer, then adjusted to an OD_600_ of 1.0. The bacterial suspensions were incubated at 28°C for 1.5 h in the dark before use. Subsequently, *in vitro*‐grown citrus seedlings were immersed in the prepared agrobacterial suspension and subjected to vacuum infiltration for 20 min. After infiltration, the seedlings were taken out, kept under moist and low‐light conditions for recovery, and then transplanted into soil for regular growth. One month after infiltration, leaf tissues were collected for total RNA extraction, and the transcript level of *CsPSMD10* was determined to evaluate gene silencing efficiency.

### Citrus hairy root transformation

For overexpression of *CsPSMD10* and its mutant *CsPSMD10‐C190S*, the full‐length coding sequences of *CsPSMD10* and *CsPSMD10‐C190S* were amplified from citrus cDNA using the specific primer pairs OE‐*CsPSMD10*‐F/OE‐*CsPSMD10*‐R and OE‐*CsPSMD10‐C190S*‐F/OE‐*CsPSMD10‐C190S*‐R, respectively, with PrimeSTAR GXL high‐fidelity DNA polymerase (TaKaRa). The amplified fragments were purified and inserted into the appropriate overexpression vector via restriction enzyme digestion and ligation, followed by verification by DNA sequencing. The validated recombinant constructs were separately transformed into *Agrobacterium rhizogenes* strain K599 harboring the pSoup helper plasmid. Positive agrobacterial colonies were cultured overnight, diluted to an optimal OD_600_ value with infiltration buffer, and used for subsequent transformation. Eureka citrus seedlings at the appropriate growth stage were selected as explants, and hairy root transformation was performed via *Agrobacterium*‐mediated infection. After co‐cultivation, the explants were transferred to rooting induction medium containing corresponding antibiotics for screening and hairy root generation. Transgenic positive hairy roots were identified by PCR and expression level detection, and the confirmed lines were used for subsequent experiments.

### Citrus transient expression and *Xcc* infiltration assays

The *SDE5640* and CsPSMD10 coding sequences were amplified using the primers pLGN‐*SDE5640*/ CsPSMD10‐F/R and cloned into the plant expression vector pLGN (Peng et al., 2017). The EHA105 single clone of pLGN‐SDE5640 was cultured in LB liquid medium containing 20 μM acetosyringone (AS). The *A*. *tumefaciens* pLGN‐ SDE5640 cells were resuspended in infiltration buffer (0.446% Murashige & Skoog salts, 0.4% sucrose, 20 mM MES, 10 mM MgCl_2_, and 150 μM AS). The OD_600_ was adjusted to 0.8, and the suspension was maintained in the dark for 1 h before infiltration. Syringes were used to inject the leaves of Wanjincheng plants. The leaves after 1 day of agroinfiltration were inoculated with Xcc to analyze the susceptibility. Before inoculating with *Xcc*, the leaves were surface‐sterilized with 75% ethanol and inoculated in an asepsis work table. Pins (0.5 mm) were used to artificially create a wound, and 1 μl of each Xcc suspension (OD_600_ c.0.8) was subsequently inoculated. After inoculation, the leaves were cultured in sterile dishes and petioles were moisturized. Lesion area and disease severity (DS) were used as resistance evaluation indexes. The following DS scoring indicators were used: 0, <0.35 mm^2^ (lesion size); 1, 0.35–0.7 mm^2^; 2, 0.7–1.05 mm^2^; 3, 1.05–1.4 mm^2^; 4, 1.4–1.75 mm^2^; 5, >1.75 mm^2^. DS was defined as disease index (DI), and the calculation formula was DI = 100 × Σ [no. (each index × corresponding index)/(total number of lesions × 5)] (He et al., 2019). The leaves were gently pierced at the injection site with an injection needle, and Xcc bacterial solution (OD_600_ = 0.8) was injected into the leaves of Wanjincheng. Orange (*C. sinensis*) expresses instantaneous infiltration, moisture, culture 7–14 days after infiltration for observation and photography (Du et al., 2023).

### Quantification of *C*Las colonization and bacterial population calculation

Total genomic DNA was isolated from citrus leaf tissues using a plant genomic DNA extraction kit, and the DNA concentration and purity were measured using a spectrophotometer. All DNA samples were adjusted to a uniform concentration for subsequent quantitative analysis. *C*Las abundance was detected by quantitative real‐time PCR (qPCR) using *C*Las‐specific primers targeting the *16S rRNA* gene. A standard curve was established using a serially diluted standard plasmid containing the *C*Las target fragment with known copy numbers. The linear regression equation of the standard curve was used to calculate the *C*Las copy number in each sample according to the corresponding Ct value. The final *C*Las population was normalized to the quantity of citrus genomic DNA and calculated with the following formula:

CLas cells μg^−1^ DNA = (CLas copy number in reaction × dilution fold)/total input DNA amount (μg). Since one copy of the *C*Las genome corresponds to one bacterial cell, the calculated copy number was directly converted to the number of *C*Las cells. The bacterial colonization level of each sample was uniformly expressed as *C*Las cell number per microgram of citrus genomic DNA (cells μg^−1^ DNA) to eliminate differences caused by DNA loading quantity and sample tissue heterogeneity.

### Western blot analysis

The total protein of the infiltrated *N. benthamiana* leaves was extracted using the Plant Total Protein Extraction Kit (CWBIO). Weigh 0.1 g benthamiana sample and absorb 500 μl protein extract, shake and mix well, then put on ice for 30 min, and then centrifuge at 13 000 rpm and 4°C for 20 min to absorb the supernatant, part of which can be used for testing and the rest can be stored at −80°C. Then, 5× loading buffer was added and boiled in a boiling water bath for 10 min, centrifuged at 12 000 rpm for 1 min, blown and mixed well. A 10 μl sample for testing was run at 80 V for half an hour, at 120 V for 2 h, and then transferred in a constant current 300 mA ice water bath for 50 min. The membranes were probed using monoclonal anti‐FLAG (Abmart), monoclonal anti‐GFP (Abmart), and anti‐HA (Abmart) antibodies, Protein IDs (Epizyme, WJ103) followed by a horseradish peroxidase‐conjugated secondary antibody (Proteintech). Then, incubated in electrochemiluminescence solution (Solarbio) for 1 min to observe the detection signal with membranes.

### Coimmunoprecipitation assay

The recombinant vectors pCV‐SDE5640‐GFP, pCV‐CsPSMD10‐Flag, pCV‐CsPSMD10‐HA, and pCV‐CsATG8s‐Flag were transferred into *A. tumefaciens* GV3101. pCV‐SDE5640‐GFP + pCV‐CsPSMD10‐Flag, pCV‐CsATG8s‐Flag + pCV‐CsATG8s‐Flag, pCV‐GFP + pCV‐CsPSMD10‐Flag, and pCV‐GFP‐HA + pCV‐CsATG8s‐Flag were co‐agroinfiltrated in *N. benthamiana* leaves. Two days later, leaves were collected co‐infiltration, and total proteins were extracted using an extraction buffer. The total proteins were incubated with GFP beads (AlpaLifeBio). The input and co‐immunoprecipitated proteins were eluted by boiling the beads in 5 × SDS loading buffer and subjected to an immunoblotting assay with anti‐GFP or anti‐Flag antibodies (Du et al., 2023).

### GST pull‐down assays

GST‐SDE5640 and His‐CsPSMD10 were introduced into BL21 (DE3) cells separately. GST‐SDE5640 and GST alone were purified using BeyoGold™GST‐tag Purification Resin (Beyotime, China), according to the manufacturer's instructions. About 1 mg of purified GST‐SDE5640 or GST alone was incubated with His‐CsPSMD10 on ice for 2 h. Subsequently, the mixture was loaded onto Glutathione Sepharose 4B resin columns. After washing five times with phosphate buffered solution with tween (PBS‐T) buffer at 4°C, the proteins were eluted from beads by boiling for 10 min in 1 × SDS sample buffer. The eluates were separated using 12% SDS‐PAGE, transferred to PVDF membranes (Millipore, USA), and probed with anti‐His (1:4000, Sigma‐Aldrich, Germany) and anti‐GST (1:50 000, Proteintech, China) antibodies. GST and His from Wuhan Genecreate (Wuhan, China) were used as negative controls. The experiments were repeated at least two times with similar results.

### MG132 treatment

Target proteins were co‐expressed in *N. benthamiana* leaves by agroinfiltration for about 24 h, then 50 μM MG132 (or DMSO for the control) was injected into the treated leaves. After 24 h, samples were punched from leaves of the same size and incubated in lysis buffer for total protein extraction, left on ice for 30 min, and centrifuged at 13 200 RPM for 30 min. The supernatant was transferred into a new 1.5 ml Eppendorf tube, mixed with 5 × SDS‐PAGE sample buffer, boiled for 10 min, and western blot analysis was performed to detect the protein expression level (Chen et al., 2020).

### Measurement of proteasome activity

This kit uses the double antibody sandwich method (Hengyuan Biological) to quantify 20S proteasome (20S PSM) levels in plant samples. Purified antibodies specific to plant 20S PSM are coated onto microporous plates, creating solid‐phase antibodies. The sample is then added to wells with monoclonal antibodies (mAb), followed by HRP‐labeled 20S PSM antibodies, forming antibody–antigen–enzyme‐labeled antibody complexes. After washing, TMB substrate is added for color development. TMB is catalyzed by HRP enzymes, initially turning blue and then yellow under acidic conditions. The intensity of this color change correlates positively with the concentration of 20S proteasome (20S PSM) in the sample. Absorbance (OD) is measured at 450 nm using an enzyme reader, and the activity of 20S proteasome (20S PSM) is calculated based on a standard curve (Mei et al., 2025).

### LC–MS/MS analysis

For LC–MS/MS‐based protein identification, *N. benthamiana* leaves were infiltrated with *A. tumefaciens* suspension carrying the recombinant pCV‐SDE5640‐GFP vector. Leaf tissues were harvested at 2 days post‐infiltration (dpi) for total protein extraction. The SDE5640‐GFP fusion protein was further enriched from crude protein extracts using GFP affinity beads, followed by protein separation via SDS‐PAGE. After bromophenol blue staining, gel segments corresponding to high‐molecular‐weight protein bands above the theoretical size of SDE5640‐GFP were excised and delivered to Oebiotech Company for liquid chromatography–tandem mass spectrometry analysis. Briefly, proteins in the collected gel fragments were subjected to trypsin digestion. The resulting peptide mixtures were fractionated by nano‐capillary high‐performance liquid chromatography and subsequently detected using a Q‐Exactive Plus mass spectrometer (Thermo Fisher, Waltham, MA, USA). For data filtering, only ubiquitination sites with a confidence expected value ≥100 were screened out and reserved for subsequent bioinformatic analysis and functional evaluation.

### Statistical analyses

The data analysis was conducted using GraphPad 8.0, and protein gray‐scale analysis was performed with ImageJ. Significant differences between experimental and control groups were determined by the two‐tailed Student's *t*‐test. Details on analytical methods, *n*‐values, and *P* values are provided in the figure legends.

## CONFLICT OF INTEREST

The authors declare that they have no conflict of interest.

## Supporting information


**Figure S1.** Molecular identification of *SDE5640*‐transgenic citrus and altered disease resistance as well as defense‐related gene expression upon *Xcc* infection.
**Figure S2.** Functional characterization of the physical interaction between SDE5640 and citrus CsPSMD10.
**Figure S3.** Molecular identification and phenotypic characterization of *CsPSMD10*‐overexpressing transgenic citrus plants and hairy roots.
**Figure S4.** The expression of genes involved in the SA‐mediated signaling pathway in *CsPSMD10* transgenic citrus.
**Figure S5.** CsPSMD10 inhibits autophagy in *Nicotiana benthamiana*.
**Figure S6.** SDE5640 activates autophagy in *Nicotiana benthamiana*.
**Figure S7.** Autophagy signaling pathway genes were repressed in the CsPSMD10‐transgenic citrus.
**Table S1.** Screening of SDE5640 interacting proteins by IP‐MS.
**Table S2.** Primers used in this study.
**Table S3.** Strains and plasmids used in this study.

## Data Availability

The data that support the findings of this study are available from the corresponding author upon reasonable request.
