# Supplementary material for: ‘Candidatus Liberibacter asiaticus’ secretory protein SDE5640 activates host autophagy dependent on PSMD10 to mediate PBA1 degradation
Source: Plant J. 2026 Oct 7;128(1):e71147. doi: 10.1111/tpj.71147 (PMC13646471; doi:10.1111/tpj.71147)
Supplement: Supplementary file 1 [file TPJ-128-0-s001.docx]

Figure S1. Molecular identification of *SDE5640*-transgenic citrus and altered disease resistance as well as defense-related gene expression upon *Xcc* infection. (A) RT-PCR verification of *SDE5640*-transgenic citrus lines. M, DL2000 DNA marker; +, positive recombinant plasmid control; −, empty vector negative control. (B) Validation of positive transgenic lines via β-glucuronidase (GUS) histochemical staining. (C) Disease symptoms of pLGN-*SDE5640* transgenic citrus leaves at 9 days post inoculation (dpi) with *Xanthomonas citri* subsp. *citri* (*Xcc*). Scale bar: 1 cm. (D, E) Quantification of *Xcc*-induced lesion sizes and disease index in transgenic leaves at 9 dpi. Error bars indicate standard errors of means, and the letters represent significant differences among samples by one-way analysis of variance followed by least significant difference post-hoc test using GraphPad Prism 8.0 (****P*< 0.001). The experiment was performed three times with similar results. (F–I) RT-qPCR analysis of pathogenesis-related and SA signaling pathway-related genes in *SDE5640*-transgenic lines. The *CsGAPC1* gene was used as an internal reference. Error bars indicate standard errors of means, and the letters represent significant differences among samples by one-way analysis of variance followed by least significant difference post-hoc test using GraphPad Prism 8.0 (****P*< 0.001). The experiment was performed three times with similar results.

**Figure S2:** Functional characterization of the physical interaction between SDE5640 and citrus CsPSMD10. (A) Phylogenetic tree analysis of PSMD10 homologous proteins derived from various plant species.(B) Y2H/Co-IP verification of the binding regions on CsPSMD10 responsible for SDE5640 interaction. SDE5640 specifically associates with the conserved ankyrin repeat domains Ank2 and Ank3 of CsPSMD10. (C) Subcellular co-localization assay showing that the interaction between SDE5640 and CsPSMD10 does not alter their subcellular distribution patterns. SDE5640-CFP and CsPSMD10-GFP fusion constructs were transiently co-expressed in Nicotiana benthamiana leaf epidermal cells by Agrobacterium-mediated infiltration. Scale bars = 50 μm. (D) Protein interaction assay demonstrating that SDE5640 is capable of binding both monomeric and homodimeric CsPSMD10.

Figure S3. Molecular identification and phenotypic characterization of *CsPSMD10*-overexpressing transgenic citrus plants and hairy roots. (A) Transcript levels of *CsPSMD10* in leaves and stems of transgenic citrus. Error bars indicate standard errors of means, and the letters represent significant differences among samples by one-way analysis of variance followed by least significant difference post-hoc test using GraphPad Prism 8.0 (*P<0.05). (B) *CsPSMD10* expression levels in healthy and HLB-infected transgenic citrus plants. Error bars indicate standard errors of means, and the letters represent significant differences among samples by one-way analysis of variance followed by least significant difference post-hoc test using GraphPad Prism 8.0 (*P<0.05). (C) Identification of positive *CsPSMD10* transgenic lines via β-glucuronidase (GUS) histochemical staining. (D) RT-PCR validation of *CsPSMD10* transgenic citrus lines. M, DL2000 DNA marker; +, positive recombinant plasmid control; −, empty vector negative control. (E) RT-PCR verification of *CsPSMD10*- and *CsPSMD10*-C190S-overexpressing transgenic citrus hairy roots. M, DL2000 DNA marker; +, positive recombinant plasmid control; −, empty vector negative control. (F) CLBV-GUS and CLBV- CsPSMD10 citrus after three months post-silencing. (G) CsPSMD10 expression was evaluated following CsPSMD10 silencing. Error bars indicate standard errors of means, and the letters represent significant differences among samples by one-way analysis of variance followed by least significant difference post-hoc test using GraphPad Prism 8.0 (*P<0.05). The experiment was performed three times with similar results. (H) Phenotypic disease symptoms of CLBV-GUS (control) and CLBV-*CsPSMD10* citrus plants after Xcc inoculation. Photographs were taken at 9 days post-inoculation (dpi). Scale bar = 10 mm. (I–J) Quantification of lesion size (I) and disease index (J) at 9 dpi in CLBV-GUS and CLBV-*CsPSMD10* citrus leaves transiently expressing pLGN-SDE5640. Error bars indicate standard errors of means, and the letters represent significant differences among samples by one-way analysis of variance followed by least significant difference post-hoc test using GraphPad Prism 8.0 (**P<0.01). The experiment was performed three times with similar results

**Figure S4:** The expression of genes involved in the SA-mediated signaling pathway in *CsPSMD10* transgenic citrus. (A–E) RT-qPCR analysis of pathogenesis-related genes and SA signaling pathway-associated genes in different transgenic citrus lines. The housekeeping gene *CsGAPC1* was used as an internal reference. Error bars represent the standard error of the mean. Error bars indicate standard errors of means, and the letters represent significant differences among samples by one-way analysis of variance followed by least significant difference post-hoc test using GraphPad Prism 8.0 (*P<0.05; ****P*< 0.001). The experiment was performed three times with similar results.

**Figure S5:** CsPSMD10 inhibits autophagy in *Nicotiana benthamiana*. (A–B) Confocal microscopic analysis of autophagic activity labeled by the autophagy marker GFP-ATG8f in *N. benthamiana* leaves. Leaves were agroinfiltrated with CFP-ATG8f together with CsPSMD10-Flag. Plants expressing GUS-Flag were used as the negative control. CFP-ATG8f-positive puncta represent autophagosomes and autophagic bodies in mesophyll cells. CFP fluorescence is shown in cyan, and chloroplast autofluorescence is shown in red. White boxes indicate the magnified regions shown in the right panels. Scale bars = 10 μm. Error bars indicate standard errors of means, and the letters represent significant differences among samples by one-way analysis of variance followed by least significant difference post-hoc test using GraphPad Prism 8.0 (*P<0.05; **P<0.01; ****P*< 0.001). The experiment was performed three times with similar results. (C) Immunoblot analysis of endogenous NBR1 protein accumulation in *N. benthamiana* leaves transiently expressing CsPSMD10-Flag.

**Figure S6**: SDE5640 activates autophagy in *N. benthamiana.* (A) Representative confocal microscopy images of autophagic activity detected by the specific autophagy marker GFP-ATG8f in *N. benthamiana* leaves infiltrated with SDE5640. Leaves infiltrated with single expression of SDE5640-GFP, GFP served as controls. Autophagosomes and autophagic bodies are revealed as CFP-positive puncta in mesophyll cells. CFP-ATG8f fusion proteins are in cyan, and chloroplasts are in red. The white boxes show the areas that are enlarged in the images on the extreme right panels. Scale bars=10 μm. (B-C) The protein level of NBR1 was detected after transient expression of SDE5640 in *N. benthamiana.* Error bars indicate standard errors of means, and the letters represent significant differences among samples by one-way analysis of variance followed by least significant difference post-hoc test using GraphPad Prism 8.0 (***P< 0.001). The experiment was performed three times with similar results. (D) Reverse transcription quantitative PCR analysis of pathogenesis related genes and CsATG8s-related genes in different transgenic lines. The CsGAPC1 gene was used as an endogenous control. Error bars indicate standard errors of means, and the letters represent significant differences among samples by one-way analysis of variance followed by LSD post hoc test (*P < 0.05; **P < 0.01).

**Figure S7:** Autophagy signaling pathway genes were repressed in the CsPSMD10-transgenic citrus. (A) Yeast two-hybrid assay showed the interaction between CsPSMD10 and CsATG7 in yeast cells,

but replace all four cysteine residues with serine residues, respectively. The results show that CsPSMD10 does not interact with ATG7. BK-53 and AD-T served as positive control, BK-53 and AD Lam served as negative control. (B-D) Reverse transcription quantitative PCR analysis of pathogenesis related genes and CsATG8s -related genes in different transgenic lines. The CsGAPC1 gene was used as an endogenous control. Error bars indicate standard errors of means, and the letters represent significant differences among samples by one-way analysis of variance followed by LSD post hoc test (*P < 0.05; **P < 0.01; ***P< 0.001). (E) The BiFc results indicate that CsATG8c does not interact with CsPBA1.

| Accession No | Sum PEP | Score Coverage | _Total Unique Peptide Count_  _GFPcontrol SDE5640_ | | Name of the Protein |
| --- | --- | --- | --- | --- | --- |
| Niben101Scf01085g02014.1 | 3 | 1 | 0 | 1 | 26S proteasome regulatory subunit 8 |
| Niben101Scf02487g00001.1 | 15 | 4 | 0 | 4 | Proteasome subunit beta 1 |
| Niben101Scf04901g00021.1 | 15 | 3 | 0 | 3 | Proteasome subunit alpha type-2-A |
| Niben101Scf07364g00017.1 | 12 | 5 | 0 | 3 | 26S proteasome non-ATPase regulatorysubunit 10 |
| Niben101Scf04549g09016.1 | 22 | 4 | 0 | 4 | Proteasome subunit beta |
| Niben101Scf04077g02006.1 | 8 | 2 | 0 | 2 | 26S proteasome non-ATPase regulatory subunit 7 homolog A |
| Niben101Scf03208g07003.1 | 3 | 3 | 0 | 3 | 26S proteasome non-ATPase regulatory subunit 1 homolog A |
| Niben101Scf02384g00024.1 | 2 | 1 | 0 | 1 | 26S proteasome non-ATPase regulatory subunit 2 homolog A |
| Niben101Scf00597g03003.1 | 11 | 3 | 0 | 3 | 26S protease regulatory subunit 8 |
| Niben101Scf09547g02002.1 | 11 | 2 | 0 | 2 | Proteasome subunit beta 2 |
| Niben101Scf01718g02010.1 | 5 | 2 | 0 | 2 | 26S proteasome non-ATPase regulatory subunit 3 homolog B |
| Niben101Scf02873g02007.1 | 5 | 1 | 0 | 1 | Proteasome subunit alpha type-7 |
| Niben101Scf02025g00002.1 | 9 | 1 | 0 | 1 | Proteasome subunit alpha type-4-2 |
| Niben101Scf09869g01020.1 | 4 | 1 | 0 | 1 | Proteasome subunit alpha type-5-B |
| Niben101Scf01052g06004.1 | 3 | 1 | 0 | 1 | 26S proteasome non-ATPase regulatory subunit 6 homolog |
| Niben101Scf10438g00008.1 | 2 | 1 | 0 | 1 | 26S proteasome non-ATPase regulatory subunit 12 homolog A |
| Niben101Scf07250g03025.1 | 3 | 1 | 0 | 1 | 26S proteasome non-ATPase regulatory subunit 4 homolog |
| Niben101Scf01848g01004.1 | 9 | 1 | 0 | 1 | Proteasome subunit beta type-3-A |
| Niben101Scf00017g00005.1 | 10 | 2 | 0 | 2 | Proteasome subunit beta 1 |
| Niben101Scf02842g01017.1 | 17 | 3 | 0 | 4 | 26S proteasome non-ATPase regulatory subunit 13 |
| Niben101Scf03488g06007.1 | 6 | 2 | 0 | 2 | Ubiquitin-associated domain-containing protein 1 |
| Niben101Scf01304g06001.1 | 4 | 2 | 0 | 2 | Ubiquitin carboxyl-terminal hydrolase 13 |
| Niben101Scf09017g00015.1 | 1 | 1 | 0 | 1 | Ubiquitin-activating enzyme E1 |
| Niben101Scf08390g06006.1 | 3 | 1 | 0 | 1 | UBX domain-containing protein 6 |
| Niben101Scf02558g00020.1 | 6 | 1 | 0 | 1 | Ubiquitin-40S ribosomal protein S27a |
| Niben101Scf12589g00003.1 | 1 | 1 | 0 | 1 | Ubiquitin carboxyl-terminal hydrolase 34 |
| Niben101Scf08680g02016.1 | 1 | 1 | 0 | 1 | Ubiquitin conjugation factor E4 |

**Table S1 Screening of SDE5640 interacting proteins by IP-MS**

|  | **Table S2 Primers used in this study** |  |
| --- | --- | --- |
| **Primer name** | **Primer sequence (5**＇**-3**＇**)** | **Connection vectors** |
| **PLGN** |  |  |
| PLGN-SDE5640-F | GGACAGGGTACCCGGGGATCCATGGATGAACCAAAGAAGCTGA | PLGN |
| PLGN-SDE5640-R | GGACAGGGTACCCGGGGATCCTTAATCACTAGATAGTTTCG |  |
| PLGN-CsPSMD10-F | GGACAGGGTACCCGGGGATCCATGGACATGGACATCGATC |  |
| PLGN-CsPSMD10-R | GGACAGGGTACCCGGGGATCCTCAACATCCGCTCCATGTCT |  |
| **RT-qPCR** |  |  |
| RT-CsPSMD10-F | AGTGTTGATGAGTCGGCGAA |  |
| RT- CsPSMD10-R | CACCACGTGAGATCAGAAGT |  |
| RT- SDE5640-F | GATGAACCAAAGAAGCTG |  |
| RT- SDE5640-R | TTAATCACTAGATAGTTTCGC |  |
| RT-CsATG8c-F | ATGGCCAAGAGCTCCTTCA |  |
| RT-CsATG8c -R | TTTGTTTTCCTCATAAATT |  |
| RT-CsATG8a-F | ATGGCCAAAAGTTCATTCA |  |
| RT-CsATG8a-R | CTTGTTTTCCTCATATATA |  |
| RT-CsATG8d-F | ATGGCGAAAAATTCTTTTA |  |
| RT-CsATG8d-R | TCAGAAGATCCCAAATGTA |  |
| RT-CsATG8f-F | ATGGCAAAGAGTTACTTCA |  |
| RT-CsATG8f-R | CTTTTTCTCTTCGTATATG |  |
| RT-CsGAPC1-F | GGAAGGTCAAGATCGGAATCAA |  |
| RT-CsGAPC1-R | CGTCCCTCTGCAAGATGACTCT |  |
| **Interaction validation** |  |  |
| pGBKT7- SDE5640-F | CATGGAGGCCGAATTCATGGATGAACCAAAGAAGCTGA | pGBKT7 |
| pGBKT7- SDE5640-R | GCAGGTCGACGGATCCTTAATCACTAGATAGTTTCGC |  |
| pGBKT7-SC2_gp095-F | TGCATATGGCCATGGAGGCCGAATTCATGGAACAAGAGAAC |  |
| pGBKT7-SC2_gp095-R | TGCGGCCGCTGCAGGTCGACGGATCCTTATTGATTAAGTGTT |  |
| pGADT7-CsPSMD10-F | GCCATGGAGGCCAGTGAATTCATGGACATGGACATCGATC | pGADT7 |
| pGADT7-CsPSMD10-R | CAGCTCGAGCTCGATGGATCCTCAACATCCGCTCCATGTCT |  |
| pGBKT7-CsATG8c-F | CATGGAGGCCGAATTCATGGCCAAGAGCTCCTTCAA |  |
| pGBKT7-CsATG8c -R | GCAGGTCGACGGATCCTTAGAAAGATGCCCCGAATGTG |  |
| pCV- SDE5640-GFP-F | TGGAGAGAACACGGGGGACTCTAGAATGGATGAACCAAAGAAGCTGA | pCV- GFP |
| pCV- SDE5640-GFP-R | TCCTCGCCCTTGCTCACCATGTCGAC TTAATCACTAGATAGTTTCGC |  |
| pCV- CsPSMD10-Flag-F | GGCGGAGGTTCTGTCGAC ATGGACATGGACATCGATC | pCV- Flag/HA |
| pCV- CsPSMD10-Flag-R | CGATCGGGGAAATTCGAGCTC TCAACATCCGCTCCATGTCT |  |
| pCV- CsATG8c-HA-F | GGCGGAGGTTCTgtcgacATGGCCAAGAGCTCCTTCAA |  |
| pCV- CsATG8c-HA-R | CGATCGGGGAAATTCGAGCTCTTAGAAAGATGCCCCGAATGTG |  |
|  |  |  |
| pCV- CsATG8a-HA-F | GGCGGAGGTTCTgtcgac ATGGCCAAAAGTTCATTCA |  |
| pCV- CsATG8a-HA-R | CGATCGGGGAAATTCGAGCTC TTAAAATGACCAACCGAAG |  |
|  |  |  |
| pCV- CsATG8d-HA-F | GGCGGAGGTTCTgtcgac ATGGCGAAAAATTCTTTTA |  |
| pCV- CsATG8d-HA-R | CGATCGGGGAAATTCGAGCTCTCAGAAGATCCCAAATGTA |  |
| pCV- CsPBA1-Flag-F | GGCGGAGGTTCTGTCGAC ATGACGAAGCAGCATCCCGC |  |
| pCV- CsPBA1-Flag-R | CGATCGGGGAAATTCGAGCTC TTAATCCTTCCTCAGTTCCA |  |
| pCV-cYFP- SDE5640-F | CTGTACAAGTCCGGAGTCGACATGGATGAACCAAAGAAGCTGA | pCV-cYFP |
| pCV-cYFP- SDE5640-R | ATCGAGGACTCCGGAGTCGACATGGATGAACCAAAGAAGCTGA |  |
| pCV-nYFP- CsPSMD10-F | ATCGAGGACTCCGGAGTCGAC ATGGACATGGACATCGATC | pCV-nYFP |
| pCV-nYFP- CsPSMD10-R | TTCGAGCTCGCCTGGGGATCC TCAACATCCGCTCCATGTCT |  |
| pCV-cYFP- CsATG8c -F | GAGCTGTACAAGTCCGGAGTCGACATGGCCAAGAGCTCCTTCAA |  |
| pCV-cYFP- CsATG8c -R | AATTCGAGCTCGCCTGGGGATCCTTAGAAAGATGCCCCGAATGTG |  |

**Table S3 Strains and plasmids used in this study**

| **Strains and vectors** | **Characteristics** | **Source** |
| --- | --- | --- |
| **Strains** |  |  |
| DH5α | *Escherichia coli strain* for plasmid construction | Shanghai Weidi Biotechnology Co.，Ltd |
| Rosetta-gami 2 (DE3) | Prokaryotic expression strain |  |
| GV3101(PJIC SA_Rep) | *Agrobacterium tumefaciens* stain |  |
| EHA105 |  |  |
| GV3101 |  |  |
| K599 | *A*. *rhizogenes* stain |  |
| Y2H Gold | *Saccharomyces cerevisiae* |  |
| **Vectors** |  |  |
| PVX | *Nicotiana benthamiana* transient expression | Lab collection |
| AD/BD | Vectors for yeast two-hybrid assays |  |
| pGEX-6P-1 | GST tag protein expression vector |  |
| pET-28a | His tag protein expression vector |  |
| CLBV | Vector for virus induced gene silencing |  |
| pLGN | Vector for overexpression | from Prof. Xiuping Zou |
| pGN | Vector for RNA interference |  |
| nLuc/cLuc | Vectors for luciferase imaging assays | from Prof. Jian-Min Zhou |
